# Ghrelin and Cannabinoid Functional Interactions Mediated by Ghrelin/CB_1_ Receptor Heteromers That Are Upregulated in the Striatum From Offspring of Mice Under a High-Fat Diet

**DOI:** 10.3389/fncel.2021.786597

**Published:** 2021-12-09

**Authors:** Alejandro Lillo, Jaume Lillo, Iu Raïch, Cristina Miralpeix, Francesc Dosrius, Rafael Franco, Gemma Navarro

**Affiliations:** ^1^Department of Biochemistry and Physiology, Faculty of Pharmacy and Food Science, University of Barcelona, Barcelona, Spain; ^2^Centro de Investigación Biomédica en Red Enfermedades Neurodegenerativas (CiberNed), National Institute of Health Carlos III, Madrid, Spain; ^3^Department of Biochemistry and Molecular Biomedicine, Universitat de Barcelona, Barcelona, Spain; ^4^Basic Sciences Department, Faculty of Medicine and Health Sciences, Universitat Internacional de Catalunya, Sant Cugat del Vallès, Spain; ^5^University of Bordeaux, INSERM, Neurocentre Magendie, Bordeaux, France; ^6^School of Chemistry, University of Barcelona, Barcelona, Spain; ^7^Institut de Neurociències, Universitat de Barcelona (UBNeuro), Barcelona, Spain

**Keywords:** CB1 cannabinoid receptor, hunger hormone, marihuana consumption, orexigenic, obesity, addiction, ghrelin receptor (GHS-R1a), cannabinoids

## Abstract

There is evidence of ghrelinergic-cannabinoidergic interactions in the central nervous system (CNS) that may impact on the plasticity of reward circuits. The aim of this article was to look for molecular and/or functional interactions between cannabinoid CB_1_ and ghrelin GHS-R1a receptors. In a heterologous system and using the bioluminescence resonance energy transfer technique we show that human versions of cannabinoid CB_1_ and ghrelin GHS-R1a receptors may form macromolecular complexes. Such receptor heteromers have particular properties in terms of CB_1_/G_i_-mediated signaling and in terms of GHS-R1a-G_q_-mediated signaling. On the one hand, just co-expression of CB_1_R and GHS-R1a led to impairment of cannabinoid signaling. On the other hand, cannabinoids led to an increase in ghrelin-derived calcium mobilization that was stronger at low concentrations of the CB_1_ receptor agonist, arachidonyl-2’-chloroethylamide (ACEA). The expression of CB_1_-GHS-R1a receptor complexes in striatal neurons was confirmed by *in situ* proximity ligation imaging assays. Upregulation of CB_1_-GHS-R1a- receptor complexes was found in striatal neurons from siblings of pregnant female mice on a high-fat diet. Surprisingly, the expression was upregulated after treatment of neurons with ghrelin (200 nM) or with ACEA (100 nM). These results help to better understand the complexities underlying the functional interactions of neuromodulators in the reward areas of the brain.

## Introduction

Cell surface cannabinoid receptors were identified as targets of natural compounds present in Cannabis sativa, specially of Δ^9^-tetrahydrocannabinol (Δ^9^-THC; (6a*R*, 10a*R*)-6,6,9-trimethyl-3-pentyl-6a,7,8,10a-tetrahydro-6H-benzo[c]chromen-1-ol; CAS registry number: #1972-08-3). So far, two cannabinoid receptors have been cloned and pharmacologically characterized, the CB_1_ and the CB_2_ receptors. They belong to class A rhodopsin-like G-protein coupled receptors (GPCRs) and both have Gi as the canonical heterotrimeric G protein to which they couple (Alexander et al., 2021). Subsequent to the discovery of cannabinoid receptors, the main compounds that act as endogenous agonists were identified, 2-arachidonoylglycerol (2-AG) and anandamide (N-arachidonoylethanolamine). Further components of the endocannabinoid system are the enzymes that synthesize and degrade 2-AG and anandamide (Lu and Mackie, 2016). Cannabis smoking leads to psychotropic events that are due to Δ^9^-THC acting on the CB_1_ receptor (CB_1_R), which is reportedly the most abundant GPCR in the central nervous system, being expressed in almost any region of the brain and both in neurons and glia (Elphick and Egertová, 2001; Mackie, 2005). In addition, it is well established that cannabis use has orexigenic properties (Pagotto et al., 2006).

Ghrelin has been considered as the “hunger hormone” (Funahashi et al., 2003; Abizaid and Horvath, 2008; Schellekens et al., 2010; Cassidy and Tong, 2017). Ghrelin, a 28-amino acid peptide produced by specialized cells of the gastrointestinal tract, activates central mechanisms that control food intake (Funahashi et al., 2003; Abizaid and Horvath, 2008; Schellekens et al., 2010; Cassidy and Tong, 2017). However, in mammals, there are overlapping mechanisms, both central and peripheral, that control food intake. Ghrelin acts *via* a specific receptor, GHS-R1a, that belongs to the family of GPCRs, couples to G_q_ heterotrimeric G protein and is expressed in a variety of cells and tissues (Pradhan et al., 2013; Alexander et al., 2019). In previous studies, we have reported physiologically relevant interactions in which the GHS-R1a receptor is involved. A functional unit composed of GHS-R1a and the dopamine D_1_ receptor mediates, at least in part, the hunger-suppressing actions of cocaine; in a macromolecular complex that also includes the non-GPCR sigma-1 receptor, a dual coupling is possible; that is, the coupling to two G proteins makes it possible for ghrelin to act through cAMP rather than through Ca^2+^ and dopamine to signal *via* increases in cytoplasmic Ca^2+^ rather than through cAMP (Casanovas et al., 2021). The ghrelin receptor is also able to interact with the CB_2_ cannabinoid receptor in both heterologous cells and in cells of the central nervous system. Cannabinoids acting on the CB_2_ receptor do not alter the cytosolic Ca^2+^ increases triggered by ghrelin. However, ghrelin receptor activation led to a blockade of CB_2_ receptor-mediated Gi-dependent signaling (Lillo et al., 2021). The aim of this article was to investigate the potential molecular and/or functional interactions between CB_1_ and GHS-R1a receptors. As the risk of obesity is higher in the progeny of obese parents, the interaction between these two receptors was also studied in neurons isolated from fetuses of mothers on a high-fat diet (Abu-Rmeileh et al., 2008).

## Materials and Methods

### Reagents

ACEA, ghrelin (human), rimonabant hydrochloride, and YIL 781 hydrochloride were purchased from Tocris Bioscience (Bristol, United Kingdom). Concentrated (10 mM) stock solutions prepared in DMSO, milli-Q^®^ H_2_O (Merck/Millipore, Darmstadt, Germany), or ethanol were stored at −20°C. In each experimental session, aliquots of concentrated solutions of compounds were thawed and conveniently diluted in the appropriate experimental solution.

### Diet-Induced Obesity Model

C57BL/6J female mice were used for the experiments. All animals were subjected to a 12 h/12 h light/ dark cycle in a temperature- and humidity-controlled room and were allowed free access to water and standard laboratory chow. C57BL/6J mice were randomly assigned to a high-fat diet (HFD; 60% kcal from fat; catalog no. D12492, Research Diets, New Brunswick, NJ, USA) or standard diet (STD; 10% kcal from fat; catalog no. D12450B, Research Diets) for 60 days. Primary striatal neurons were obtained from fetuses of mothers on STD or HFD diets. Pregnant animals were killed by cervical dislocation during the light phase. All animal procedures were performed in agreement with European guidelines (2010/63/EU) and approved by the University of Barcelona Ethical Committee, which reports to the regional Government (Protocol #9659; Generalitat de Catalunya, May 24, 2019).

### Cell Culture and Transient Transfection

Human embryonic kidney HEK-293T (lot 612968) cells were acquired from the American Type Culture Collection (ATCC). They were amplified and frozen in liquid nitrogen in several aliquots. Cells from each aliquot were used until passage 19. HEK-293T cells were grown in Dulbecco’s modified Eagle’s medium (DMEM; Gibco, Paisley, Scotland, United Kingdom) supplemented with 2 mM L-glutamine, 100 U/ml penicillin/streptomycin, MEM Non-Essential Amino Acid Solution (1/100), and 5% (v/v) heat-inactivated Fetal Bovine Serum (FBS; all supplements were from Invitrogen, Paisley, Scotland, United Kingdom). Cells were maintained in a humid atmosphere of 5% CO_2_ at 37°C.

Cells were transiently transfected with the corresponding cDNAs using the PEI (PolyEthylenImine, Sigma-Aldrich, St. Louis, MO, USA) method as previously described (Carriba et al., 2008; Hradsky et al., 2011; Navarro et al., 2012). Four hours after transfection, growth medium was replaced by a complete medium. Experiments were carried out 48 h later.

To prepare primary striatal neurons, brains from fetuses of pregnant mice were removed (gestational age: 17 days). Neurons were isolated as described in Hradsky et al. (2013) and plated at a confluence of 40,000 cells/0.32 cm^2^. Briefly, the samples were dissected and, after careful removal of the meninges, digested for 20 min at 37°C with 0.25% trypsin. Trypsinization was stopped by adding an equal volume of culture medium (Dulbecco’s modified Eagle medium-F-12 nutrient mixture, Invitrogen). Cells were brought to a single cell suspension by repeated pipetting followed by passage through a 100 μm-pore mesh. Pelleted (7 min, 200× *g*) cells were resuspended in supplemented DMEM and seeded at a density of 3.5 × 10^5^ cells/ml. The next day, the medium was replaced by neurobasal medium supplemented with 2 mM L-glutamine, 100 U/ml penicillin/streptomycin, and 2% (*v*/*v*) B27 medium (Gibco). Neuronal cultures were used for assays after 15 days of culture. Using NeuN as a marker, the percentage of neurons in the cultures was >90%.

### Expression Vectors

The human cDNAs for the CB_1_R, GHS-R1a, and D_1_R cloned in pcDNA3.1 were amplified without their stop codons using sense and antisense primers. The primers harbored either unique BamHI and KpnI sites for CB_1_R and HindIII and BamHI sites for GHS-R1a and D_1_R. The fragments were subcloned to be in frame with an enhanced yellow fluorescent protein (pEYFP-N1; Clontech, Heidelberg, Germany) and the Renilla luciferase protein (Rluc; pRluc-N1; PerkinElmer, Wellesley, MA) on the C-terminal end of the receptor to produce CB_1_R-YFP, D_1_R-Rluc, and GHS-R1a-Rluc.

### Immunofluorescence

HEK-293T cells transfected with cDNAs for CB_1_R-YFP and GHS-R1a-Rluc were fixed in 4% paraformaldehyde for 15 min and then washed twice with PBS containing 20 mM glycine before permeabilization with the same buffer containing 0.2% Triton X-100 (5 min incubation). The samples were treated for 1 h with blocking solution (PBS containing 1% bovine serum albumin) and labeled with a mouse anti-Rluc (1/100; MAB4400, Millipore, Burlington, MA, USA) as primary antibody and subsequently treated with Cy3-conjugated anti-mouse IgG (1/200; 715-166-150; Jackson ImmunoResearch) as the secondary antibody (1 h each). The samples were washed several times and mounted with 30% Mowiol (Calbiochem, San Diego, USA). Nuclei were stained with Hoechst (1/100). Samples were observed under a Zeiss 880 confocal microscope (Carl Zeiss, Oberkochen, Germany).

### Bioluminescence Resonance Energy Transfer (BRET) Assay

HEK-293T cells growing in 6-well plates were transiently cotransfected with a constant amount of cDNA encoding for GHS-R1a fused to Renilla luciferase (GHS-R1a-Rluc) and with increasing amounts of cDNA corresponding to CB_1_ receptor fused to the yellow fluorescent protein (CB_1_R-YFP). For negative control, cells were cotransfected with a constant amount of cDNA encoding for D_1_R-Rluc and with increasing amounts of cDNA for CB_1_R-YFP. Forty-eight hours post-transfection cells were washed twice in quick succession with HBSS (137 mM NaCl; 5 mM KCl; 0.34 mM Na_2_HPO_4_; 0.44 mM KH_2_PO_4_; 1.26 mM CaCl_2_; 0.4 mM MgSO_4_; 0.5 mM MgCl_2_; and 10 mM HEPES, pH 7.4) supplemented with 0.1% glucose (w/v), detached by gently pipetting and resuspended in the same buffer. To have an estimation of the number of cells per plate, protein concentration was determined using a Bradford assay kit (Bio-Rad, Munich, Germany) with bovine serum albumin dilutions for standardization. To quantify YFP-fluorescence expression, cells were distributed (20 μg protein) in 96-well microplates (black plates with a transparent bottom; Porvair, Leatherhead, UK). Fluorescence was read using a Mithras LB 940 (Berthold, Bad Wildbad, Germany) equipped with a high-energy xenon flash lamp, using a 10-nm bandwidth excitation and emission filters at 485 and 530 nm, respectively. YFP-fluorescence expression was determined as the fluorescence of the sample minus the fluorescence of cells expressing only protein-Rluc. For the BRET measurements, the equivalent of 20 μg of cell suspension was distributed in 96-well microplates (white plates; Porvair), and 5 μM coelenterazine H was added (PJK GMBH, Kleinblittersdorf, Germany). Then, 1 min after coelenterazine H addition, the readings were collected using a Mithras LB 940 (Berthold, Bad Wildbad, Germany), which allowed the integration of the signals detected in the short-wavelength filter at 485 nm (440–500 nm) and the long-wavelength filter at 530 nm (510–590 nm). To quantify receptor-Rluc expression, luminescence readings were collected 10 min after 5 μM coelenterazine H addition. The net BRET is defined as [(long-wavelength emission)/(short-wavelength emission)]-Cf where Cf corresponds to [(long-wavelength emission)/(short-wavelength emission)] for the Rluc construct expressed alone in the same experiment. The BRET curves were fitted assuming a single phase by a non-linear regression equation using the GraphPad Prism software (San Diego, CA, USA). BRET values are given as milli-BRET units (mBU: 1000 × net BRET).

### cAMP Determination

HEK-293T cells transfected with the cDNAs for CB_1_R (1 μg) and/or GHS-R1a (1.5 μg) and neuronal primary cultures were plated in 6-well plates. Two hours before initiating the experiment, the cell-culture medium was replaced by the non-supplemented DMEM medium. Then, cells were detached, resuspended in the non-supplemented DMEM medium containing 50 μM zardaverine, and plated in 384-well microplates (2,500 cells/well). Cells were pretreated (15 min) with the corresponding antagonists (1 μM rimonabant for CB_1_R and 1 μM YIL 781 for GHS-R1a) or vehicle and stimulated with agonists (1 nM, 10 nM, 100 nM, and 1 μM ACEA for CB_1_R or 200 nM ghrelin for GHS-R1a; 15 min) before the addition of 0.5 μM FK or vehicle. Finally, the reaction was stopped by the addition of the Eu-cAMP tracer and the ULight-conjugated anti-cAMP monoclonal antibody prepared in the “cAMP detection buffer” (PerkinElmer). All steps were performed at 25°. Homogeneous time-resolved fluorescence energy transfer (HTRF) measures were performed after 60 min incubation at RT using the Lance Ultra cAMP kit (PerkinElmer, Waltham, MA, USA). Fluorescence at 665 nm was analyzed on a PHERAstar Flagship microplate reader equipped with an HTRF optical module (BMG Lab Technologies, Offenburg, Germany).

### MAPK Phosphorylation Assays

To determine extracellular signal-regulated kinase 1/2 (ERK1/2) phosphorylation, HEK-293T transfected cells and primary striatal neurons were plated (50,000 cells/well) in transparent Deltalab 96-well plates and kept in the incubator for 15 days. Two hours before the experiment, the medium was replaced by non-supplemented DMEM medium. Next, the cells were pre-treated at RT for 10 min with antagonists (1 μM rimonabant for CB_1_R and YIL 781 for GHS-R1a) or vehicle and stimulated for an additional 7 min with selective agonists (1 nM, 10 nM, 100 nM, 1 μM ACEA for CB_1_R and 200 nM ghrelin for GHS-R1a). Then, cells were washed twice with cold PBS before the addition of 30 μl/well “Ultra lysis buffer” -PerkinElmer- (15 min treatment). Afterward, 10 μl of each supernatant was placed in white ProxiPlate 384-well plates and ERK1/2 phosphorylation was determined using an AlphaScreen^®^SureFire^®^ kit (PerkinElmer), following the instructions of the supplier, and using an EnSpire^®^ Multimode Plate Reader (PerkinElmer, Waltham, MA, USA). The reference value (100%) was the value achieved in the absence of any treatment (basal). Agonist effects were given in percentage with respect to the basal value.

### Real-Time Determination of Calcium Ion Cytoplasmic Level Variation

HEK-293T cells were transfected with the cDNAs for CB_1_R (1 μg) and/or GHS-R1a (1.5 μg) in the presence of 1 μg cDNA for the calmodulin-based calcium GCaMP6 sensor (Chen et al., 2013) using the PEI method. 48 h after transfection, cells were detached using Mg^+2^-free Locke’s buffer (pH 7.4; 154 mM NaCl, 5.6 mM KCl, 3.6 mM NaHCO_3_, 2.3 mM CaCl_2_, 5.6 mM glucose and 5 mM HEPES) supplemented with 10 μM glycine. 1,500 cells per well were plated in 96-well black, clear-bottom, microtiter plates. Then, cells were incubated for 10 min with the CB_1_R and GHS-R1a antagonists (1 μM rimonabant or 1 μM YIL 781), and subsequently stimulated with selective agonists (1 nM, 10 nM, 100 nM, 1 μM ACEA, or 200 nM ghrelin). Upon excitation at 488 nm, real-time 515 nm fluorescence emission due to calcium-ion complexed GCaMP6 was recorded on the EnSpire^®^ Multimode Plate Reader (every 5 s, 100 flashes per well).

### Proximity Ligation Assays (PLAs)

Physical interaction between CB_1_R and GHS-R1a was detected using the Duolink *in situ* PLA detection Kit (OLink; Bioscience, Uppsala, Sweden) following the instructions of the supplier. Primary neurons were grown on glass coverslips, fixed in 4% paraformaldehyde for 15 min, washed with PBS containing 20 mM glycine to quench the aldehyde groups, and permeabilized with the same buffer containing 0.05% Triton X-100 (20 min). Then, samples were successively washed with PBS. After 1 h incubation at 37°C with the blocking solution in a pre-heated humidity chamber, primary neurons were incubated overnight in the antibody diluent medium with a mixture of equal amounts of mouse anti-CB_1_R (1/100; sc-293419, Santa Cruz Technologies, Dallas, TX, USA) and rabbit anti-GHS-R1a (1/100; ab95250, Abcam, Cambridge, United Kingdom) to detect CB_1_R-GHS-R1a complexes. Neurons were processed using the PLA probes detecting primary antibodies (Duolink II PLA probe plus and Duolink II PLA probe minus) diluted in the antibody diluent solution (1:5). Ligation and amplification were done as indicated by the supplier. Samples were mounted using the mounting medium with Hoechst (1/100; Sigma-Aldrich) to stain nuclei. Samples were observed in a Zeiss 880 confocal microscope (Carl Zeiss, Oberkochen, Germany) equipped with an apochromatic 63× oil immersion objective (N.A. 1.4) and a 405 nm and a 561 nm laser lines. For each field of view, a stack of two channels (one per staining) and four Z stacks with a step size of 1 μm were acquired. The number of neurons containing one or more red spots vs. total cells (blue nucleus) was determined, and the unpaired t-test was used to compare the values (red dots/cell) obtained.

## Results

### The CB_1_R May Interact With the GHS-R1a

**Figure 1:**
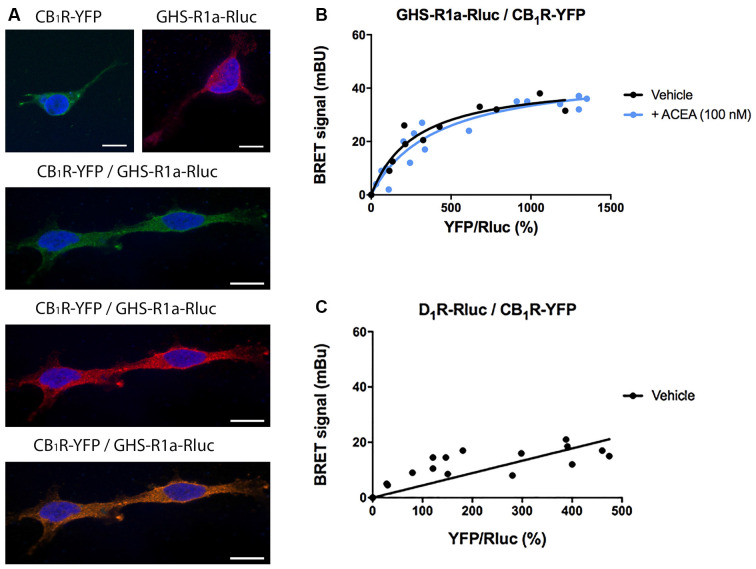
Molecular interaction between GHS-R1a and CB_1_ receptors expressed in HEK-293T cells. **(A)** Confocal microscopy images of HEK-293T cells transfected with cDNAs for GHS-R1a-Rluc (1.5 μg) and/or for CB_1_R-YFP (1 μg). GHS-R1a-Rluc (red) was identified by immunocytochemistry using an anti-Rluc antibody (Merck-Millipore, 1/100). The CB_1_R-YFP (green) was identified by the fluorescence due to YFP. Colocalization is shown in yellow. Cell nuclei were stained with Hoechst (blue). Scale bar: 10 μm. **(B,C)** BRET saturation experiments were performed using HEK-293T cells co-transfected with a constant amount of GHS-R1a-Rluc cDNA (1.5 μg) and increasing amounts of CB_1_R-YFP cDNA (0–2 μg) and treated with ACEA (100 nM) or vehicle. As a negative control, HEK-293T cells were transfected with a constant amount of D_1_R-Rluc cDNA (1.5 μg) and increasing amounts of CB_1_R-YFP cDNA (0–2 μg). BRET data are expressed as the mean ± SEM of eight independent experiments performed in duplicates. mBU: milliBRET units.

*Cannabis sativa L* consumption has an orexigenic effect *via* a mechanism in which a hormone of the endocrine system, ghrelin, participates. To identify whether or not there are functional interactions between the cannabinoidergic and the orexinergic systems, we first tested a potential interaction between the CB_1_R and the functional form of the ghrelin receptor, GHS-R1a. Immunocytochemical assays in HEK-293T cells transfected with the cDNA of the GHS-R1a fused to Renilla luciferase (Rluc) and/or the cDNA for the CB_1_R fused to the Yellow Fluorescent Protein (YFP) led to detect the receptors at the plasma membrane level with a marked colocalization when coexpressed (Figure 1A).

As colocalization may be found for proteins that are close (approximately 200 nm apart) but may not be directly interacting, a Bioluminescence Resonance Energy Transfer (BRET) assay was performed in HEK-293T cells cotransfected with a constant amount of the cDNA for GHS-R1a-Rluc and increasing amounts of cDNA for CB_1_R-YFP. A saturation curve (BRET_max_ = 44 ± 4 mBU, BRET_50_ = 280 ± 70) was obtained, demonstrating a direct interaction between the two receptors in this heterologous expression system (Figure 1B). When the same experiment was performed in cells pretreated for 30 min with the selective CB_1_R agonist, arachidonyl-2’-chloroethylamide (ACEA, 100 nM), no significant differences were observed (BRET_max_ = 47 ± 4 mBU, BRET_50_ = 400 ± 10). This result indicates that CB_1_R-GHS-R1a interaction is not affected by the activation of the CB_1_R. As a negative control, HEK-293T cells were transfected with a constant amount of dopamine D_1_ receptor-Rluc cDNA and increasing amounts of CB_1_R-YFP cDNA; the nonspecific linear signal indicates a lack of interaction between these two proteins (Figure 1C).

### CB_1_R-Mediated Signaling Is Blocked in the CB_1_-GHS-R1a Receptor Heteromer (CB_1_R-GHS-R1aHet)

**Figure 2:**
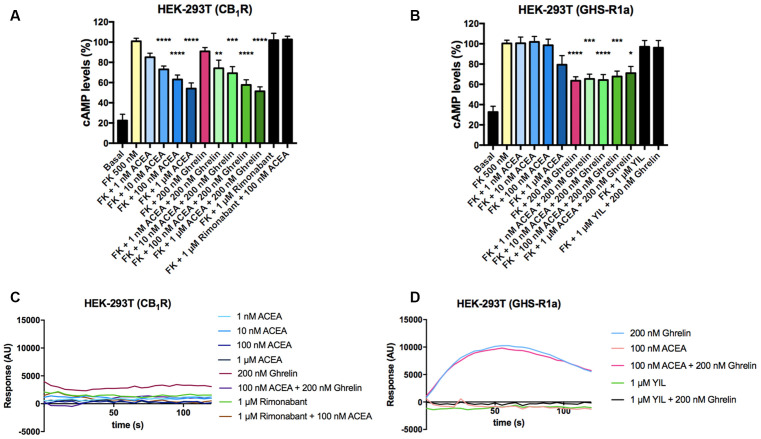
Functional characterization of GHS-R1a and CB_1_ receptors expressed in HEK-293T cells. **(A,B)** HEK-293T cells transfected with cDNA encoding for either CB_1_R (1 μg; **A**) or GHS-R1a (1.5 μg; **B**) were pre-treated with selective antagonists, 1 μM rimonabant -CB_1_R- or 1 μM YIL 781 -GHS-R1a-, and subsequently treated with the selective agonists, ACEA (1 nM, 10 nM, 100 nM, 1 μM) -CB_1_R- or ghrelin (200 nM) -GHS-R1a-. cAMP levels after 0.5 μM forskolin (FK) stimulation were detected by the Lance Ultra cAMP kit. Results are expressed in % respect to levels obtained upon FK stimulation (100%). The values are the mean ± SEM of 10 independent experiments performed in triplicates. One-way ANOVA followed by Bonferroni’s multiple comparison *post-hoc* test were used for statistical analysis. *p < 0.05, **p < 0.01, ***p < 0.001, ****p < 0.0001 vs. FK treatment. **(C,D)** HEK-293T cells expressing an engineered calcium sensor, 6GCaMP, and CB_1_R **(C)** or GHS-R1a **(D)** were pre-treated with selective antagonists for 10 min followed by agonist stimulation. Real-time traces of cytoplasmic Ca^2+^ levels detected by EnSpire^®^ Multimode Plate Reader (PerkinElmer, Waltham, MA, USA) over time are shown (six independent experiments).

After identifying a direct interaction between CB_1_R and GHS-R1a, the functional consequences of the interaction were investigated. Signaling assays were performed considering that the CB_1_R couples to G_i_ and that, although the canonical protein that couples to GHS-R1a receptor is G_q_, the ghrelin receptor may also couple to G_i_. The activation of any of the receptors in the presence of forskolin (FK), which activates adenylate cyclase, led to a decrease in cytosolic cAMP levels in HEK-293T cells expressing CB_1_R or GHS-R1a. In cells expressing the CB_1_R, the selective agonist, arachidonyl-2’-chloroethylamide (ACEA), led to a dose-response decrease in (FK)-stimulated cAMP levels that were not affected by pretreatment with ghrelin. Moreover, ghrelin treatment induced no effect over cannabinoid CB_1_R, demonstrating ligand selectivity. The effect of ACEA was specific as it was blocked by a selective CB_1_R antagonist, rimonabant (Figure 2A). In cells expressing the GHS-R1a, 200 nM ghrelin induced a significant (circa 30%) decrease of FK-induced cAMP level that was not affected by ACEA but that was completely counteracted by YIL 781, a selective GHS-R1a receptor antagonist (Figure 2B). It was confirmed that ACEA did not induce any effect over the GHS-R1a. When the G_q_ coupling was assayed using the GCaMP6 sensor of cytoplasmic Ca^2+^, no signal was obtained in cells expressing the CB_1_R (Figure 2C), while in cells expressing the GHS-R1a receptor ghrelin lead to a transient peak of cytosolic [Ca^2+^] that was not modified by preincubation with ACEA but that was prevented upon preincubation with the ghrelin receptor antagonist (Figure 2D).

**Figure 3:**
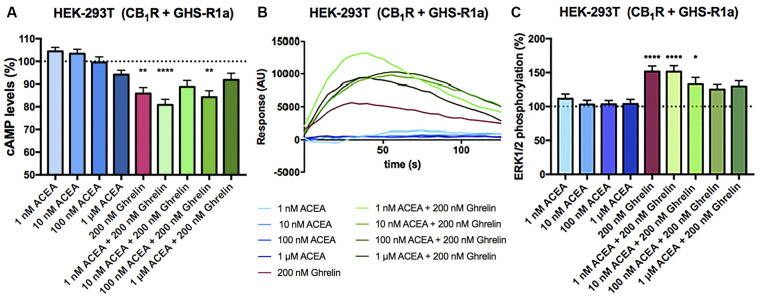
Functional characterization of the CB_1_-GHS-R1aHet expressed in HEK-293T cells. **(A–C)** HEK-293T cells were transfected with cDNAs encoding for GHS-R1a (1.5 μg) and for CB_1_R (1 μg; **A,C**) or cDNAs encoding for GHS-R1a (1.5 μg), CB_1_R (1 μg) and the 6GCaMP calcium sensor (1 μg; **B**) and stimulated with selective agonists, 200 nM ghrelin -for GHS-R1a- and 1 nM, 10 nM, 100 nM, and 1 μM ACEA -for CB_1_R-, individually or in combination. cAMP levels were analyzed by the Lance Ultra cAMP kit and results are expressed in % respect to levels obtained upon 0.5 μM FK stimulation (**A**; 100%, dotted line). Representative traces of intracellular Ca^2+^ responses over time are shown (eight independent experiments; **B**). ERK 1/2 phosphorylation was determined by an AlphaScreen^®^SureFire^®^ kit (PerkinElmer) using an EnSpire^®^ Multimode Plate Reader (PerkinElmer, Waltham, MA, USA; **C**); (basal is represented as 100%, dotted line). In cAMP accumulation and MAPK pathway signaling-related assays, the values are the mean ± SEM of eight independent experiments performed in triplicates. One-way ANOVA followed by Bonferroni’s multiple comparison *post-hoc* test were used for statistical analysis. *p < 0.05, **p < 0.01, ****p < 0.0001; vs. FK treatment in cAMP or vs. basal in ERK1/2 phosphorylation assays.

In HEK-293T cells expressing CB_1_ and GHS-R1a receptors, the ghrelin-induced decrease of FK-stimulated cAMP levels was significant although lower than that in cells only expressing the ghrelin receptor (37% vs. 15%; Figure 3A). Interestingly, the presence of the ghrelin receptor uncoupled CB_1_R activation from G_i_ protein, at least at a functional level. In fact, ACEA even at the highest concentration (1 μM) was not able to significantly reduce FK-induced cAMP levels. Functionality was, however, found when cells were treated with ghrelin and there was no additive effect at concentration of 10 nM ACEA or higher; the lower concentration of ACEA (1 nM) showed a trend to increase the ghrelin-induced effect (Figure 3A). Results related to G_q_ coupling were noteworthy as ACEA, which did not lead to calcium ion mobilization by itself, significantly increased the effect of ghrelin. Remarkably, the potentiation was much stronger at low doses of ACEA (1 nM) than at higher concentrations (Figure 3B). In particular, the 40 s post-activation increase provided by the presence of 1 nM, 10 nM or 100 nM ACEA over the signal provided by 200 nM ghrelin, was (in percentage), 140 ± 30, 65 ± 30, and 71 ± 15, respectively. Finally, as GHS-R1a and CB_1_ receptor agonists lead to activation of the mitogen-activated protein kinase (MAPK) pathway (Mousseaux et al., 2006; Daigle et al., 2008; Navarro et al., 2018b), we tested the properties of the heteromer in the link to the MAPK signaling pathway. Again, the effect of the cannabinoid receptor agonist was suppressed when the two receptors were co-expressed, while the effect of ghrelin was not modified by low doses of ACEA but was diminished when higher doses were used (Figure 3C). In a control assay, it was confirmed that ACEA does increase ERK1/2 phosphorylation, as previously reported (Navarro et al., 2018a, b). These data demonstrate that cannabinoids may regulate GHS-R1a function depending on the concentration and that GHS-R1a expression suppresses cannabinoid receptor-mediated events supposedly by the establishment of heteromeric complexes.

### Cross-Talk Characterization

In the 90s different laboratories proved interactions between GPCRs to form heteromeric complexes. These complexes can be detected by energy transfer techniques in heterologous expression systems or by proximity ligation assays (PLA) in natural sources, either primary cultures or tissue sections. An often-found property of a heteromer formed by two GPCRs is that the antagonist of one of the receptors not only blocks the signaling originated at the receptor but also the signaling originated at the partner receptor. Such cross-antagonism, which is due to conformational changes transmitted from one receptor to the other, may serve as a print to detect the heteromer. Another possibility is that coactivation leads to a smaller effect than that obtained upon activating only one receptor of the complex; this phenomenon is known as negative crosstalk. Finally, in some cases, the antagonist of one receptor may restore the signaling *via* the partner receptor in the heteromer.

**Figure 4:**
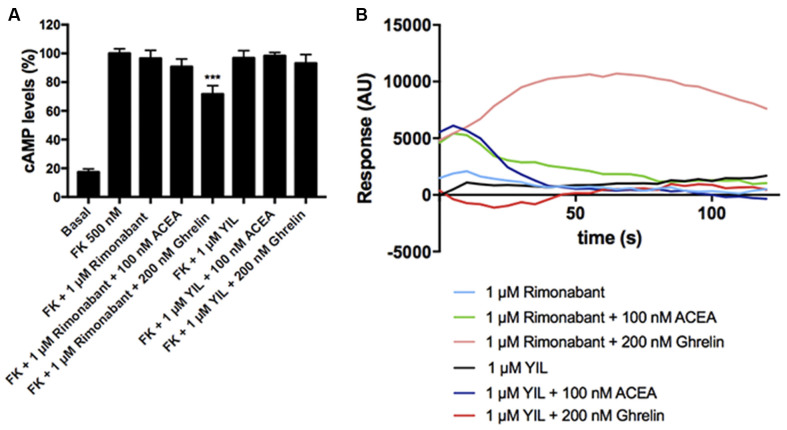
Effect of antagonists in the functionality of the CB_1_-GHS-R1aHet. HEK-293T cells were transfected with cDNAs encoding for GHS-R1a (1.5 μg) and for CB_1_R (1 μg; **A**) or with cDNAs encoding for GHS-R1a (1.5 μg), CB_1_R (1 μg), and the 6GCaMP calcium sensor (1 μg; **B**). Cells were pre-treated with antagonists, 1 μM YIL 781 -for GHS-R1a- or 1 μM rimonabant -for CB_1_R-, and subsequently stimulated with selective agonists, 200 nM ghrelin -for GHS-R1a- or 100 nM ACEA -for CB_1_R-. cAMP levels were analyzed by the Lance Ultra cAMP kit and results were expressed in % respect to levels obtained upon 0.5 μM FK stimulation (**A**; 100%). Representative real- time traces of cytoplasmic Ca^2+^ responses are shown (eight independent experiments; **B**). In cAMP accumulation assays, the values are the mean ± SEM of eight independent experiments performed in triplicates. One-way ANOVA followed by Bonferroni’s multiple comparison *post-hoc* test were used for statistical analysis. ***p < 0.001; vs. FK treatment.

To study the effect of antagonists, HEK-293T cells expressing CB_1_ and GHS-R1a receptors were pretreated with the selective antagonists before agonist stimulation. As observed in Figure 4A, the CB_1_R antagonist, rimonabant, did not block G_i_-mediated ghrelin-induced effect, whereas the GHS-R1a antagonist, YIL 781, which completely blocked ghrelin-induced decrease of FK-induced cAMP levels, did not restore the CB_1_R-G_i_ coupling. Accordingly, there was no cross-antagonism in GHS-R1a/G_i_-mediated signaling when the CB_1_ receptor was blocked by a selective antagonist. Similar were results in Figure 4B, i.e., there was no cross-antagonism in GHS-R1a receptor/G_q_-mediated signaling when the CB_1_R receptor was blocked by a selective antagonist. In summary, neither cross-antagonism nor restoration of CB_1_R-G_i_ coupling was observed when addressing direct G_i_- or direct G_q_-induced outputs using selective antagonists.

### Effect of Ghrelin and/or ACEA in Primary Striatal Neurons

**Figure 5:**
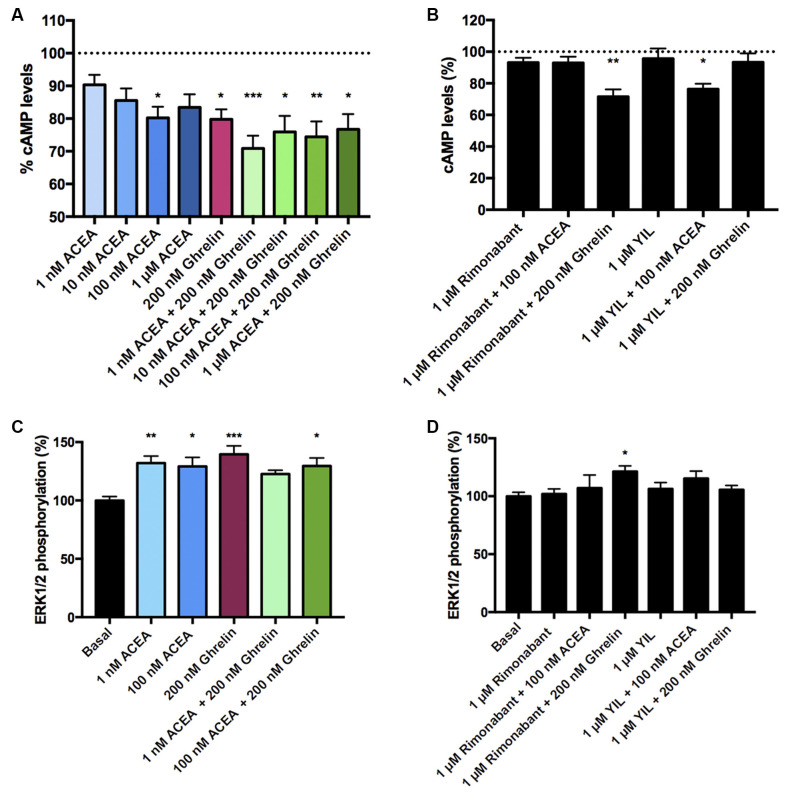
CB_1_R-GHS-R1aHet signaling in primary striatal neurons from C57BL/6J mice. Primary striatal neurons obtained from C57BL/6J brain fetuses were pre-treated with selective antagonists, 1 μM YIL 781 -for GHS-R1a- or 1 μM rimonabant -for CB_1_R- or vehicle and subsequently stimulated with selective agonists, 200 nM ghrelin -for GHS-R1a- or 1 nM, 10 nM, 100 nM, and 1 μM ACEA -for CB_1_R-, individually or in combination. cAMP levels **(A,B)** were collected by the Lance Ultra cAMP kit and results are expressed in % respect to levels obtained upon 0.5 μM FK stimulation (100%, dotted line). ERK1/2 phosphorylation **(C,D)** was analyzed using an AlphaScreen^®^SureFire^®^ kit (PerkinElmer) and results are expressed in % respect to basal levels. Values are the mean ± SEM of six independent experiments performed in triplicates. One-way ANOVA followed by Bonferroni’s multiple comparison *post-hoc* tests were used for statistical analysis. *p < 0.05, **p < 0.01, ***p < 0.001; vs. FK treatment **(A,B)** or vs. basal **(C,D)**.

After demonstrating the functionality of CB_1_R-GHS-R1aHet in transfected HEK-293T cells, we moved to a more physiologic environment to look for the expression of the receptor complex. We investigated the expression and function of CB_1_R-GHS-R1aHet in primary striatal neurons. First, we analyzed the effect of ACEA and ghrelin on FK-induced cAMP. As observed in Figure 5, ACEA was able to induce a dose-dependent effect. Ghrelin also decreased the FK-induced cAMP levels. Coactivation with ghrelin and 1 nM ACEA resulted in an additive effect, but not at higher ACEA concentrations. Moreover, the effect of ACEA was not affected by the antagonists of the GHS-R1a receptor and, reciprocally, the effect of ghrelin was not counteracted by the antagonists of the CB_1_R (Figure 5B). On the other hand, when analyzing ERK1/2 phosphorylation in striatal primary neurons, both ACEA and ghrelin led to a significant increase in phosphorylation levels. In addition, no cross-antagonism was detected in primary neurons pretreated with rimonabant followed by ghrelin stimulation (Figures 5C,D). These results suggest that the proportion of CB_1_R-GHS-R1aHets in primary striatal cultures is relatively low and that CB_1_ and GHS-R1a receptors may be forming complexes with other GPCRs (see “Discussion” section).

### The CB_1_R-GHS-R1aHet Is Overexpressed in Striatal Neurons Isolated From the Brain of the Progeny of Mothers Under a High-Fat Diet

**Figure 6:**
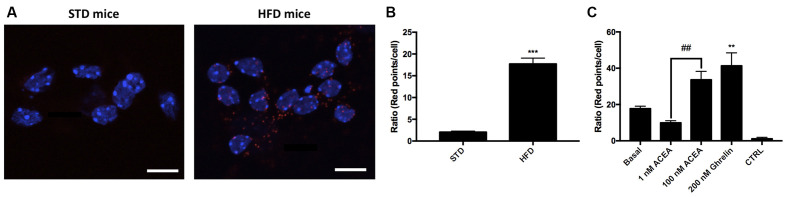
Expression and function of CB_1_-GHS-R1aHets in primary striatal neurons isolated from fetuses of pregnant C57BL/6J female mice subjected to a standard (STD) or high-fat (HFD) diet. **(A)** CB_1_-GHS-R1aHets were detected by the *in situ* proximity ligation assay (PLA) in primary striatal neurons isolated from the striatum of fetuses from STD or HFD pregnant mothers. Experiments were performed in samples from six animals. **(B,C)** The number of red dots/cell was quantified using Andy’s algorithm Fiji’s plug-in; nuclei were stained using Hoechst (blue). In **(B)** the number of dots/cell in HFD samples are compared with that in STD samples. In **(C)** neurons obtained from fetuses from HFD mothers were stimulated with 200 nM ghrelin -for GHS-R1a- or 1 nM and 100 nM ACEA -for CB_1_R- and analyzed by PLA **(C)**. The negative control was obtained omitting one of the primary antibodies. Scale bar: 10 μm. One-way ANOVA followed by Bonferroni’s multiple comparison *post-hoc* test was used for statistical analysis on comparing to basal or to STD: ***p < 0.001, vs. STD **(B)** **p < 0.01, vs. basal **(C)**. Bonferroni’s multiple comparison test showed significance in comparing results from ACEA treatments: ^##^*p* < 0.01 **(C)**.

One of the aims of this study was to correlate the expression of CB_1_R-GHS-R1a receptor complexes in a situation of unbalanced energy homeostasis. For this purpose, we used primary striatal neurons isolated from fetuses of mothers fed a standard (STD) or high fat (HFD) diet (see “Materials and Methods” section). The expression of CB_1_R-GHS-R1aHets was assessed by *in situ* proximity ligation assay (PLA). An important increase in the expression of CB_1_-GHS-R1a complexes was observed in striatal neurons from fetuses of HFD mothers (18 red dots/cell vs. 3 red dots/cell in neurons from fetuses of STD mothers; Figures 6A,B). Moreover, when primary neurons from fetuses of HFD mothers were treated with 1 nM ACEA, a significant decrease in complex expression (12 red dots/cell) was observed; however, a higher concentration of the compound, 100 nM, led to a marked increase in the number of complexes (36 red dots/cell). Remarkably, treatment with 200 nM ghrelin produced a robust increase in CB_1_R-GHS-R1a complex expression (>40 red dots/cell; Figure 6C). In summary, the expression of the CB_1_R-GHS-R1aHet was higher in the progeny of HFD mothers and may be regulated by ghrelin and, differentially, by cannabinoid concentration.

## Discussion

Cannabis has been known and used for years by various civilizations and is used even today. Cannabis use is perceived in two almost opposite ways. One is related to the psychotropic properties of one of its components, (6a*R*, 10a*R*)-6,6,9-trimethyl-3-pentyl-6a,7,8,10a-tetrahydro-6H-benzo[c]chromen-1-ol, commonly known as THC (or Δ^9^THC; CAS registry number #1972-08-3). It is under question whether the exposure to THC is addictive although it is known that the compound affects homeostatic synaptic plasticity. The good side is the possibility that compounds in *Cannabis sativa L* may be useful to combat a variety of diseases. For instance, dronabinol (Marinol^®^), with an identical chemical structure to THC but of synthetic origin, has been already approved for human use. Also approved are a preparation having an equal amount of THC and cannabidiol (Sativex^®^) and a preparation of pure cannabidiol in vegetable oil (Epidiolex^®^). The main therapeutic indications of these drugs are anti-emetic and spasticity management. Interestingly, a CB_1_R antagonist, rimonabant, was approved to combat obesity. The discovery that blockade of the CB_1_R could lead to a drop in body weight by reducing food consumption correlated with the well-known orexigenic properties of Cannabis consumption. Unfortunately, the compound, which is brain permeable, was withdrawn due to serious adverse events related to alterations in central functions. Although it was hypothesized that CB_1_R antagonists unable to cross the blood-brain barrier could overcome the side effects, there is evidence that the anti-orexigenic actions of CB_1_R antagonists are due to, at least in part, the blockade of receptors in the central nervous system where the CB_1_ is the most abundant GPCR (Carai et al., 2006; Christensen et al., 2007; Sam et al., 2011; Tudge et al., 2015). In this scenario, we aimed at defining possible interactions between cannabinoid receptors and the receptor for the so-called “hunger” hormone, ghrelin. In a recent article, we have shown the interaction of the cannabinoid CB_2_ receptor (CB_2_R) with the GHS-R1a receptor in a heterologous expression system and in physiological cell models (Lillo et al., 2021). Therefore, the first aim of the present study was to address the possible interaction between the ghrelin receptor and the CB_1_R and to characterize the functional consequences of such interaction. Both, the previous and the present studies demonstrate that the GHS-R1a receptor may interact with either CB_1_ or CB_2_ receptors and that the resulting heteromers may occur in physiological environments.

At the functional level, allosteric interactions within CB_1_R-GHS-R1aHets and of CB_2_R-GHS-R1aHets lead to the blockade of cannabinoid receptor/G_i_-mediated signaling. The blockade occurs just by simple co-expression, i.e., it does not require the activation of the GHS-R1a receptor. Taking these results together, cells expressing cannabinoid and ghrelin receptors heteromers on the cell surface would not be responsive to cannabinoids, or even to endocannabinoids, unless there is a pool of cell surface CB_1_Rs that are not interacting with the ghrelin receptor.

Several phytocannabinoids are able to cross the blood-brain barrier (Sagredo et al., 2007; Lafuente et al., 2011; Espejo-Porras et al., 2013; García et al., 2016; Libro et al., 2016; Palomo-Garo et al., 2016; Zeissler et al., 2016; Valdeolivas et al., 2017; Haider et al., 2018; Franco et al., 2020) and reach the brain reward circuits in which ghrelin acts. Ghrelin receptors in reward circuits mediate the control of food intake (Guan et al., 1997; Funahashi et al., 2003; Geelissen et al., 2003; Argente-Arizón et al., 2015; Cassidy and Tong, 2017). Our data indicate a blockade of G_i_/CB_1_R coupling but a potentiation induced by cannabinoids of the GHS-R1a/G_q_-mediated events, namely a potentiation in calcium-mediated signaling. In our opinion, the higher potentiation at low doses of the CB_1_R agonist (1 nM) is physiologically relevant. It is tempting to speculate that the atypical effect depending on the dose, i.e., higher potentiation at lower doses underlies previous results in humans and mice showing that low doses of THC are associated with hyperphagia, whereas high doses suppress it (Simon and Cota, 2017). As previously highlighted, endocannabinoid tone may be important in controlling the inputs received by the reward circuits and that impact on food intake, especially as it relates to the hedonic part of eating (Coccurello and Maccarrone, 2018). Whereas cannabinoids acting on the CB_1_ receptor affected calcium mobilization mediated by GHS-R1a-G_q_ coupling, this is not the case for the CB_2_R-GHS-R1aHet (Lillo et al., 2021). As further discussed below, caution must be taken when trying to make general conclusions as the allosteric-cross interactions will occur in neurons expressing CB_1_R and GHS-R1a receptors and also CB_1_R-GHS-R1aHets; i.e., not all neurons express the two receptors, and a given neuron may express the CB_1_R-GHS-R1aHet plus other CB_1_R-containing heteromers (see, in http://www.gpcr-hetnet.com/, GPCRs that interact with the CB_1_R; accessed on October 22, 2021).

As the risk to be obese is higher in families with a history of overweighed individuals, we reasoned that the expression of the heteromer could be altered in the offspring of high-fat-diet mouse mothers as they have more risk to be obese. Compared with samples from fetuses of mothers subjected to STD, there was a marked increase of CB_1_R-GHS-R1aHets expression in striatal neurons from siblings of pregnant female mice under a high-fat diet. Such an increase in heteromer expression might be implicated in the obesity predisposition of the progeny of obese parents (Abu-Rmeileh et al., 2008). Upregulation of the CB_1_R-GHS-R1aHet in siblings of mothers fed with HFD suggests that already at birth, these mice have a compromised CB_1_R function. In addition, we observed that the number of heteromers markedly increased by activation of either the CB_1_ with 100 nM ACEA or the GHS-R1a receptors with ghrelin. The upregulation induced by ACEA treatment in neurons did not occur in transfected HEK-293T cells, where ACEA pretreatment does not alter the BRET saturation curve. Also noteworthy is the fact that treatments are short, i.e., changes in expression are not due to regulation of gene expression but to conformational rearrangements within the receptor-receptor and receptor-G protein interactions. Another piece of information is that, in our hands, the CB_1_R/G_i_-mediated effect of 100 nM ACEA observed in striatal primary neurons was completely blocked in transfected HEK-293T cells expressing the CB_1_R-GHS-R1aHet. This result indicates that not all striatal neurons express the heteromer and/or that the CB_1_R in a given neuron may be interacting with receptors other than the GHS-R1aHet, i.e., with receptors that are not allosterically blocking CB_1_R-mediated signaling. In the so called “*hedonic eating*” by Coccurello and Maccarrone (2018), dopamine plays a key role in the reward circuits. Accordingly, dopamine receptors may be considered in the overall picture. In this sense, the CB_1_R may interact with dopamine receptors; it has been reported that the cannabinoid receptor may, at least, interact with the dopamine D_2_ receptor (Jarrahian et al., 2004; Navarro et al., 2008; Marcellino et al., 2008; Przybyla and Watts, 2010; Khan and Lee, 2014; Köfalvi et al., 2020), reviewed in García et al. (2016). Cannabinoid CB_1_/dopamine D_2_ receptor-receptor interaction is bidirectional and may result in functional antagonism, i.e., a CB_1_R agonist blocking the D_2_R-mediated modulation of locomotor activity (Marcellino et al., 2008) or in a shift from G_i_ to G_s_ coupling and signaling (Bagher et al., 2017).

In conclusion, the benefits of cannabinoids acting on populations of striatal neurons expressing CB_1_R-GHS-R1aHet would be lost by the blockade exerted by the ghrelin receptor, thus prevailing the effect of such cannabinoids on other systems such as the dopaminergic. On the other hand, the CB_1_R-GHSR1aHet coactivated by ghrelin and cannabinoids provides a more robust calcium response. Such bursts in the concentration of cytosolic calcium must be relevant since calcium regulates almost any event of neuronal physiology. Our results suggest the potential for GHS-R1a receptor antagonists, which could offer a double benefit: (i) reduce food intake and (ii) revert the detrimental effects of HFD on the functionality of the CB_1_R in striatal neurons.

It is quite likely that the CB_1_R-GHSR1aHet does occur in given subpopulations of neurons. Therefore, the next stage would be an accurate description, at the anatomical and cellular levels, of the regions and specific neurons where the two receptors are co-expressed.

## Data Availability Statement

The raw data supporting the conclusions of this article will be made available by the authors, without undue reservation.

## Ethics Statement

The animal study was reviewed and approved by University of Barcelona Ethical Committee, which reports to the regional Government (Protocol #9659; Generalitat de Catalunya, May 24, 2019).

## Author Contributions

GN and RF: conceptualization, supervision, and writing—original draft. RF: data curation. AL, JL, and GN: formal analysis. AL, JL, IR, FD, CM, and GN: investigation, writing—review and editing. AL, JL, and IR: methodology. AL: project administration. CM, GN, and RF: resources. GN: validation. All authors have read and agreed to the published version of the manuscript. All authors contributed to the article and approved the submitted version.

## Conflict of Interest

The authors declare that the research was conducted in the absence of any commercial or financial relationships that could be construed as a potential conflict of interest.

## Publisher’s Note

All claims expressed in this article are solely those of the authors and do not necessarily represent those of their affiliated organizations, or those of the publisher, the editors and the reviewers. Any product that may be evaluated in this article, or claim that may be made by its manufacturer, is not guaranteed or endorsed by the publisher.
